# The first case of *Francisella novicida* infection in Taiwan: bacteraemic pneumonia in a haemodialysis adult

**DOI:** 10.1080/22221751.2022.2026199

**Published:** 2022-01-21

**Authors:** Hao-En Jan, Chin-Shiang Tsai, Nan-Yao Lee, Pei-Fang Tsai, Li-Rong Wang, Po-Lin Chen, Wen-Chien Ko

**Affiliations:** a Department of Internal Medicine, National Cheng Kung University Hospital, College of Medicine, National Cheng Kung University, Tainan, Taiwan; b Department of Internal Medicine, National Cheng Kung University Hospital, Dou-Liou Branch, College of Medicine, National Cheng Kung University, Yunlin, Taiwan; c Institute of Clinical Medicine, College of Medicine, National Cheng Kung University, Tainan, Taiwan; d Center for Infection Control, National Cheng Kung University Hospital, College of Medicine, National Cheng Kung University, Tainan, Taiwan; e Department of Pathology, National Cheng Kung University Hospital, College of Medicine, National Cheng Kung University, Tainan, Taiwan; f Department of Medicine, College of Medicine, National Cheng Kung University, Tainan, Taiwan

**Keywords:** *Francisella novicida*, bacteraemia, pneumonia, 16S ribosomal RNA, Taiwan

## Abstract

Tularaemia is a zoonotic disease caused by *Francisella tularensis* (*F. tularensis*). Human infection is rare and can be life-threatening. *F. tularensis subsp. novicida* used to be a subspecies of *F. tularensis*, is now considered a different species, *F. novicida*. Though less virulent, *F. novicida* can cause morbidity and mortality among debilitated or immunocompromised patients. We reported that an adult with end-stage renal disease undergoing haemodialysis and a history of melioidotic aortic aneurysm developed *F. novicida* bacteraemic pneumonia, which was uneventfully treated by antimicrobial therapy. The microbiological confirmation of *F. novicida* infection relies on 16S rRNA sequencing. It is the first case of *F. novicida* infection in Taiwan.

Tularemia is a zoonotic disease caused by *Francisella tularensis* (*F. tularensis*), which has been regarded as one of the bioweapons. Human infection is rare but can be life-threatening [1]. *F. tularensis* comprises three subspecies: *tularensis* (type A), *holarctica* (type B), and *mediasiatica*. The former two subspecies, *tularensis* and *holarctica,* are responsible for tularaemia in humans [2, 3]. *F. tularensis* subsp. *novicida* used to be a subspecies of *F. tularensis*, and is now considered a different species, *F. novicida* [4]. In contrast, *F. novicida* is less virulent, but can cause morbidity and mortality among debilitated or immunocompromised patients [5, 6]. Here we presented a successfully managed case of *F. novicida* bacteraemia.

A 63-year-old man with underlying hypertension and end-stage renal disease undergoing haemodialysis three times per week was admitted to a tertiary hospital in southern Taiwan. He presented with one-week history of intermittent fever, chills, malaise, and mild productive cough. He recalled the consumption of sashimi one week before the onset of fever but denied the contact of animals or contaminated dust, insect bites, or oral ingestion of unboiled water. Notably, two years ago he had experienced recurrent bacteraemia and mycotic thoracic aortic aneurysm caused by *Burkholderia pseudomallei*, which were successfully treated with parenteral antibiotic for 9 months and thoracic aortic replacement.

On admission, his body temperature was 38°C, blood pressure 148/80 mmHg, heart rate 114 beats/minute, and respiratory rate 20 breaths/minute. Physical examination found two small non-ulcerative lymph nodes over the right submandibular and inguinal areas, respectively. Crackles were auscultated on left lung field. Laboratory exams revealed leukocyte count 11,200/mm^3^, neutrophil 87.1%, lymphocyte 7.8%, haemoglobin 8.7 g/dl, and platelet count 366,000/mm^3^. Serum electrolyte and liver functions were within normal limits. Serum erythrocyte sedimentation rate was 140 mm/h (normal: <15 mm/h), ferritin 1,153 ng/ml (normal: 30–400 ng/ml), iron 14 μg/dl (normal: 33–193 μg/dl), total iron-binding capacity 94 μg/dl (normal: 245–419 μg/dl). The chest x-ray film and computed tomography revealed patchy consolidation of bilateral lower lungs, especially left lung. His serum anti-*Mycoplasma pneumoniae* immunoglobulin M (IgM) and sputum cultures showed negative results.

Since recurrent melioidosis was considered, meropenem and doxycycline were initiated, and fever and cough improved on the second day. Serial chest radiographs noted the resolution of pulmonary consolidation (Figure 1). Gram-negative bacilli were yielded from blood cultures collected upon admission. Microscopically, the bacteria were faintly stained, Gram-negative, rarely chained pleomorphic coccobacilli. The isolate was able to grow on a blood agar plate at 35°C. The biochemical phenotype of the isolate was oxidase-negative, catalase-positive (weak), and glucose non-fermenting. VITEK MS (bioMérieux, Marcy l’Etoile, France) failed to identify the pathogen, and the VITEK2 GN ID card (bioMérieux, Durham, NC, USA) reported a low-level discrimination between *Pseudomonas fluorescens* and *Sphingomonas paucimobilis*. The metabolizing abilities of this isolate detected by the VITEK2 GN ID card were summarized in the Table 1.

**Figure 1.**
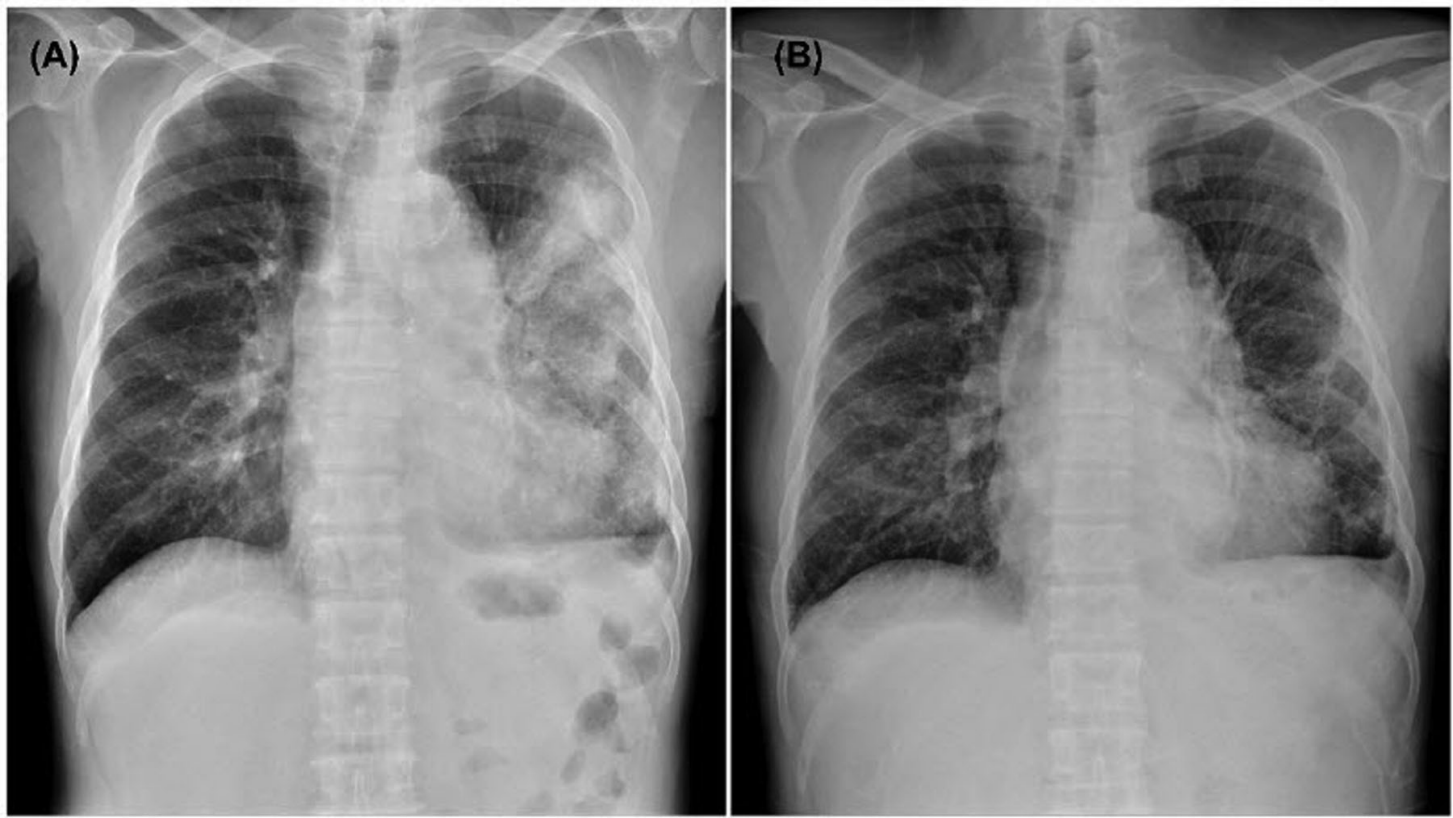
Chest plain films show patchy infiltrates and dense consolidation in the left lung upon admission (A) and apparent resolution of consolidation in the left lung on hospital day 15 (B).

**Table 1.** Metabolizing abilities of the present *Francisella novicida* strain and two Gram-negative bacillary species.

Biochemistry tests	*F. novicida* strain in this report	*Pseudomonas fluorescens*	*Sphingomonas paucimobilis*
Ala-Phe-Pro-arylamidase	(−)	(−)	(+)
Adonitol	(−)	(−)	(−)
L-pyrrolydonyl-arylamidase	(+)	(+)	(−)
L-arabitol	(−)	(−)	(−)
D-cellobiose	(−)	(−)	(+)
Beta-galactosidase	(−)	(−)	(−)
H_2_S production	(−)	(−)	(−)
Beta-N-acetyl-glucosaminidase	(−)	(−)	(−)
Glutamyl arylamidase pNA	(−)	(−)	(−)
D-glucose	(−)	(+)	(+)
Gamma-glutamyl-transferase	(+)	(−)	(+)
Fermentation/glucose	(−)	(−)	(−)
Beta-glucosidase	(−)	(−)	(−)
D-maltose	(−)	(−)	(+)
D-mannitol	(−)	(−)	(−)
D-mannose	(−)	(−)	(−)
Beta-xylosidase	(−)	(−)	(−)
Beta-alanine arylamidase pNA	(−)	(−)	(−)
L-proline arylamidase	(+)	(+)	(+)
Lipase	(−)	(−)	(−)
Palatinose	(−)	(−)	(−)
Tyrosine arylamidase	(+)	(+)	(+)
Urease	(−)	(−)	(−)
D-sorbitol	(−)	(−)	(−)
Saccharose/sucrose	(−)	(−)	(−)
D-tagatose	(−)	(−)	(−)
D-trehalose	(−)	(−)	(−)
Citrate (sodium)	(−)	(−)	(−)
Malonate	(−)	(−)	(−)
5-keto-D-gluconate	(−)	(−)	(−)
L-lactate alkalinization	(−)	(+)	(−)
Alpha-glucosidase	(−)	(−)	(−)
Succinate alkalinization	(+)	(+)	(+)
Beta-N-acetyl-galactosaminidase	(−)	(−)	(−)
Alpha-galactosidase	(−)	(−)	(−)
Phosphatase	(+)	(−)	(+)
Glycine arylamidase	(−)	(−)	(−)
Ornithine decarboxylase	(−)	(−)	(−)
Lysine decarboxylase	(−)	(−)	(−)
Decarboxylase base	(−)	(−)	(−)
L-histidine assimilation	(−)	(−)	(−)
Courmarate	(−)	(−)	(−)
Beta-glucoronidase	(−)	(−)	(−)
O/129 resistance (comp.vibrio.)	(−)	(−)	(−)
Glu-Gly-Arg-arylamidase	(−)	(−)	(−)
L-malate assimilation	(−)	(−)	(−)
Ellman	(−)	(−)	(−)
L-lactate assimilation	(−)	(−)	(−)

The species identification of *F. novicida* was made by 16S rRNA polymerase chain reaction (PCR) assay. Two primers, 11F (GTTTG-ATCCT-GGCTC-AG) and 1512R (GGYTA-CCTTG-TTACG-ACTT), were used to amplify the conserved 16S rRNA genes. Two identical 1,050 base-pair amplicons were obtained. Using the GenBank database, two amplified fragments were 99.4% identical to the genes of *F. novicida* strain AZ06-7470 (GenBank accession number: CP009682). The agar gradient dilution test using E-test (AB Biodisk, Solna, Sweden) found it was susceptible to ciprofloxacin (minimal inhibitory concentration: 0.032 μg/mL), levofloxacin (0.032 μg/mL), and tetracycline (0.75 μg/mL), based on the clinical breakpoints of *F. tularensis* proposed by the Clinical and Laboratory Standards Institute (CLSI) [7]. After 9 days of meropenem therapy and 14 days of doxycycline, he was discharged without major sequelae.

*F. tularensis* was first isolated in 1912 as the causative agent of a plague-like disease affecting squirrels in California [8], and can cause human diseases, tularaemia, most common in the south central United States, the Pacific Northwest, and parts of Massachusetts, including Martha’s Vineyard [3]. *F. tulrarensis* subsp. *tularensis* is localized in North America and *F. tulrarensis* subsp. *holarctica* is found throughout the Northern Hemisphere and in Australia [5, 9, 10]. In comparison, *F. novicida,* a closely related subspecies of *F. tularensis*, rarely causes illness in immunocompromised individuals and has not been isolated from animals. Despite varying human pathogenicity, *F. tularensis* and *F. novicida* share 97% nucleotide identity [5]. Therefore, precise species identification is critical for accurate interpretation of experimental results and ensuring treatment efficacy studies utilizing virulent *F. tularensis* strains [5].

Typical clinical patterns of tularemia, including ulceroglandular, glandular, oropharyngeal, oculoglandular, or gastrointestinal manifestations, have not been reported for *F. novidica* infection. Up to now, there is no evidence for human-to-human transmission of *F. novicida* infection. Several cases of *F. novicida* infection presenting with cervical lymphadenitis, bacteremia, or skin and soft-tissue infection with inguinal lymphadenitis, have been reported [11–14.] Of note, in the English literature, there was only one case of *F. novicida* infection reported in Asia [13]. In our case, bacteremic pneumonia was clinically evident and was not associated with the previous illness of melioidosis-related mycotic aneurysm. However, the development of community-onset bacteremia illness due to two uncommon Gram-negative bacilli suggests an immunocompromised status in this patient, which is likely related to end-stage renal disease.

Clinical manifestations of *F. novicida* infections in previous reports usually were non-specific and the pathogen might be misidentified. Therefore, the clinical impact of *F. novicida* infections may be underestimated. Human may acquire *F. tularensis* by bites of vector arthropods, direct contact of infected animals, ingestion of food or water contaminated by infected animals, or inhalation of infected aerosols. On the contrary, *F. novicida* is likely an aquatic bacterium and has been linked to waterborne transmission [5, 15]. For all cases of human *F. novicida* infection with an identified mode of transmission, an aquatic source was always involved. For example, *F. novicida* infection has occurred in a patient with a near-drowning accident [14, 16, 17]. However, *F. novicida* has not been identified in naturally infected animals [5, 17, 18]. Notably, there has been an unusual cluster of three cases at a correctional facility in Louisiana linked to contaminated ice as the potential vehicle of transmission [14]. A previous study demonstrated that by *sdhA* sequencing *Francisella*-like isolates from the soil had a closer genetic relationship with the subspecies *novicida* [19]. So far, this is the first autochthonous case of *F. novicida* infection in Taiwan, but the acquisition route of *F. novicida* remains unknown after thorough queries of the history of travelling abroad or contact with animals or raw water. However, the patient mentioned the ingestion of raw seafood (sashimi); therefore, the likelihood of *F. novicida* transmitted by ingestion of uncooked fish cannot be excluded.

In conclusion, we report the first case of *F. novicida* infection in Taiwan, which presented as bacteremic pneumonia with a previous history of melioidosis mycotic aneurysm. Microbiological confirmation of *F. novicida* infection relies on 16S rRNA sequencing. Future investigations are warranted to elucidate the transmission route and local epidemiology of *F. novicida* in Taiwan.
